# Clinicopathological characteristics and prognostic factors in young patients after hepatectomy for hepatocellular carcinoma

**DOI:** 10.1186/1477-7819-11-52

**Published:** 2013-03-02

**Authors:** Shingo Shimada, Toshiya Kamiyama, Hideki Yokoo, Kenji Wakayama, Yosuke Tsuruga, Tatsuhiko Kakisaka, Hirofumi Kamachi, Akinobu Taketomi

**Affiliations:** 1 Department of Gastroenterological Surgery I, Hokkaido University Graduate School of Medicine, Kita15-Nishi7, Kita-Ku, 060-8638, Sapporo, Hokkaido, Japan

**Keywords:** Hepatocellular carcinoma, Young, Hepatectomy, Clinicopathological characteristics, Prognostic factors

## Abstract

**Background:**

The aim of this study was to analyze the clinicopathological characteristics and the prognostic factors for survival and recurrence of young patients who had undergone hepatectomy for hepatocellular carcinoma.

**Methods:**

Between 1990 and 2010, 31 patients aged 40 years or younger (younger patient group) among 811 consecutive patients with hepatocellular carcinoma who had undergone primary hepatectomy were analyzed with regard to patient factors, including liver function, tumor factors and operative factors. The clinicopathological characteristics of the younger patients were compared with those of patients over the age of 40 (older patient group). Then the prognostic factors of the younger patients were analyzed. Continuous variables were expressed as the means ± standard deviation and compared using the χ^2^ test for categorical variables. Overall survival and recurrence-free survival rates were determined by the Kaplan-Meier method and analyzed by the log-rank test. The Cox proportional hazards model was used for multivariate analysis.

**Results:**

In the younger patients, the rates of HBs-antigen-positivity, high alpha-fetoprotein, portal invasion, intrahepatic metastasis, large tumors, low indocyanin green retention rate at 15 minutes, and anatomical resection were significantly higher than the same measures in the older patients. The five-year overall survival rate of the young patients was 49.6%. The prognostic factors of survival were HCV-antibody-positivity and low albumin status. Prognostic factors of recurrence were multiple tumors and the presence of portal invasion.

**Conclusions:**

In younger patients, survival appeared to be primarily affected by liver function, while recurrence was affected by tumor factors. Young patients with hepatocellular carcinoma should be aggressively treated with hepatectomy due to their good pre-surgical liver function.

## Background

Liver cancers are malignant tumors and are the third leading cause of cancer-related death; they are responsible for approximately 700,000 deaths per year [1]. Hepatocellular carcinoma (HCC) has a poor prognosis and accounts for 70 to 85% of primary liver cancers [2]. Generally, there are few opportunities for discovery of malignant tumors in younger patients, and thus they tend to present with a highly advanced malignancy at the time of diagnosis; nonetheless, younger patients can expect long-term survival. The definition of what constitutes a “young patient” differs between studies [3-12]. HCC is fairly rare in younger individuals, with an occurrence rate of only 0.6 to 2.7% in those under 40 years of age, according to Japanese reports [12-14]. In Asia and Africa, which are areas with prevalent hepatitis B virus (HBV), the frequency of HCC is higher than in Japan [4,8,9,11,15]; however, there are still few reports on independent prognostic factors in young patients with HCC.

In this study, we examined the prognostic clinicopathological features, as well as the prognostic factors for survival and recurrence, in young patients with HCC who had undergone hepatectomy.

## Methods

Between January 1990 and May 2010, 811 consecutive patients with HCC underwent primary liver resection at the Gastroenterological Surgery I unit of Hokkaido University Hospital in Sapporo, Japan. Of these patients, 31 patients (3.8%) were 40 years old or younger, while 780 patients (96.2%) were over 40 years of age. For group stratification, the former patients were defined as the younger patient group, and the latter as the older patient group. This study was approved by the Hokkaido University Hospital Voluntary Clinical Study Committee and was performed according to the Helsinki Declaration guidelines. The clinicopathological characteristics and surgical data of the patients are shown in Table 1.

**Table 1:** Clinicopathological characteristics

	**Young (age ≤40 years)**	**Old (age >40 years)**	***P***
	**n = 31**	**n = 780**	
Epidemiology			
Sex: Male/Female	24/7 (77%/23%)	644/136 (83%/17%)	*NS*
HBs-Ag positive	26 (84%)	321 (41%)	<0.0001
HCV-Ab positive	1 (3%)	310 (40%)	<0.0001
Biochemical Factors			
Albumin ≥4.0 g/l	17 (55%)	411 (53%)	*NS*
Total bilirubin ≥0.8 mg/dl	17 (55%)	379 (49%)	*NS*
ICGR15 ≥15	3 (10%)	360 (46%)	0.0001
AFP ≥200 ng/ml	16 (52%)	210 (27%)	0.0026
Tumor Factors			
Number of tumors: 1	20 (65%)	522 (67%)	*NS*
2 to 3	6 (19%)	183 (23%)	
≥4	5 (16%)	75 (10%)	
Maximum size of tumors: <2 cm	4 (12%)	83 (11%)	0.0074
≥2 cm, <5 cm	7 (23%)	395 (50%)	
≥5 cm	20 (65%)	303 (39%)	
Macroscopic classification: simple nodular type	10 (32%)	408 (52%)	*NS*
simple nodular type with extranodular grow	10 (32%)	222 (28%)	
confluent multinodular type	8 (26%)	122 (16%)	
infiltrative type	0 (0%)	6 (1%)	
others	3 (10%)	22 (3%)	
Distant metastasis positive	2 (6%)	18 (2%)	*NS*
Surgical Factors			
Anatomical resection	29 (94%)	525 (67%)	0.0021
Histological Factors			
Differentiation: well	3 (10%)	114 (15%)	*NS*
moderate	13 (42%)	430 (55%)	
poor	14 (45%)	209 (27%)	
others	1 (3%)	27 (3%)	
vp:vp0	14 (45%)	569 (73%)	0.0026
vp1	9 (29%)	125 (16%)	
vp2,3,4	8 (26%)	86 (11%)	
im	16 (52%)	264 (34%)	0.0413
cirrhosis	9 (29%)	287 (37%)	*NS*

AFP, alpha-fetoprotein; HBs-Ag, HBs-antigen; HCV-Ab, HCV-antibody; ICGR15, indocyanin green retention rate at 15 minutes; im, microscopic intrahepatic metastasis; NS, non-significant; vp0, no tumor thrombus in the portal vein; vpl, tumor thrombus distal to the second branches of the portal vein; vp2, tumor thrombus in the second branches of the portal vein; vp3, tumor thrombus in the first branch of the portal vein; vp4, tumor thrombus extension to the trunk or the opposite side branch of the portal vein.

The indications for hepatic resection and the type of operative procedures were usually determined based on the patients’ liver function reserve, that is, according to the results of the indocyanin green retention test at 15 minutes (ICGR15) [16]. Anatomical resection was performed on patients in whom the ICGR15 was lower than 25%. Anatomical resection was defined as a resection in which the lesions were completely removed anatomically on the basis of Couinauds’ classification (segmentectomy, sectionectomy, and hemihepatectomy or more). Non-anatomical partial but complete resection was achieved in other cases. In all patients, surgery was performed at R0 or R1. When R0 and R1 resections were performed, the resection surfaces were found to be histologically or macroscopically free of HCC, respectively. Follow-up studies after liver resection were conducted at three-month intervals, which included physical, serological (liver function test, serum alpha-fetoprotein (AFP) level, and serum protein induced by vitamin K absence-II (PIVKA-II)), and radiological examinations (ultrasound sonography (US) and contrast-enhanced computed tomography (CT) scan or contrast-enhanced magnetic resonance imaging (MRI)). Recurrence was diagnosed on the basis of the results of contrast-enhanced CT and elevation of serum levels of AFP and/or PIVKA-II. Extrahepatic metastasis (lung, lymph node, adrenal gland, brain and bone) was diagnosed by contrast-enhanced chest and abdominal CT, contrast-enhanced head MRI and bone scintigram. The median follow-up period was 111 months (range, 5 to 249 months).

### Statistical analysis

Continuous variables were expressed as the means ± standard deviation and compared using the χ^2^ test for categorical variables. Overall survival (OS) and recurrence-free survival (RFS) were determined by the Kaplan-Meier method and analyzed by the log-rank test. The Cox proportional hazards model was used for multivariate analysis. Significance was defined as a *P*-value of <0.05. Statistical analyses were performed using Stat View 5.0 for Windows (SAS Institute, Cary, NC, USA).

## Results

### Clinicopathological characteristics and operative variables

#### Patient factors

The ratio of males to females (24:7) in the younger patient group was not significantly different from that of the older patient group. Patients with HBV markers accounted for most of the virus-associated cases: HBs-antigen (HBs-Ag)-positive, 26/31 (total number in the younger group) vs. 321/780 (total number in the older group); 84% vs. 41%; *P* <0.0001. Patients who were hepatitis C virus (HCV)-antibody (HCV-Ab)-positive were significantly fewer in number, that is, 1/31 vs. 310/780 (3% vs. 40%; *P* <0.0001) in the younger group. Although serum albumin and total bilirubin levels were not significantly different between the groups, patients with ICGR15 ≥15 were 3/31 vs. 360/780 (10% vs. 46%; *P* = 0.0001).

#### Tumor factors

The younger group had significantly higher AFP levels compared to the older group (*P* = 0.0026). Although the number of tumors did not differ significantly between the younger and older patients, there were significantly more cases with a maximum tumor size of ≥5 cm in the younger group (*P* = 0.0072). The mean maximum tumor diameter in the younger group in this study was 8.6 ± 7.3 cm. Neither macroscopic type nor extrahepatic metastasis was significantly different between the groups.

#### Operative variables

The rate of anatomical resections in the younger patients was significantly higher than that in the older patients.

#### Pathological factors

There were significant differences between groups in terms of microscopic tumor thrombus in the portal vein (*P* = 0.0026) and microscopic intrahepatic metastasis (*P* = 0.0413) (Table 1).

### Causes of death and recurrence

Among the total 811 patients, 390 (48.1%) died. The mortality rates were 17/31 (54.8%) in the younger patient group and 373/780 (47.8%) in the older patient group. The causes of death, which did not differ significantly between groups, were as follows: HCC recurrence (n = 301; 77.2%; 16 in the younger patients vs. 285 in the older patients), liver failure (n = 36; 9.2%; 0 in the younger vs. 36 in the older patients), and other causes (n = 53; 13.6%; 1 in the younger vs. 52 in the older patients). In addition, two patients in the older group died of operative complications prior to 1995. No patients in the younger group died of operative complications.

In the younger group, 22 patients experienced a recurrence (71.0%). There were 17 (77.3%) liver tumor recurrences, with a median recurrence time of six months (1 to 27). Lung metastases occurred in 11 (50.0%) cases, with a median recurrence time of 12 months (1 to 42); bone metastases in 7 (31.8%) cases, with a median recurrence time of 23 months (6 to 60); brain metastases in 6 (27.3%) cases, with a median recurrence time of 20 months (10 to 61); lymph node metastases in 3 (13.6%) cases, with a median recurrence time of 12 months (12 to 56); and adrenal gland metastases in 3 (13.6%) cases, with a median recurrence time of 10 months (5 to 50).

### Cumulative rates of patient survival and recurrence-free survival

**Figure 1:**
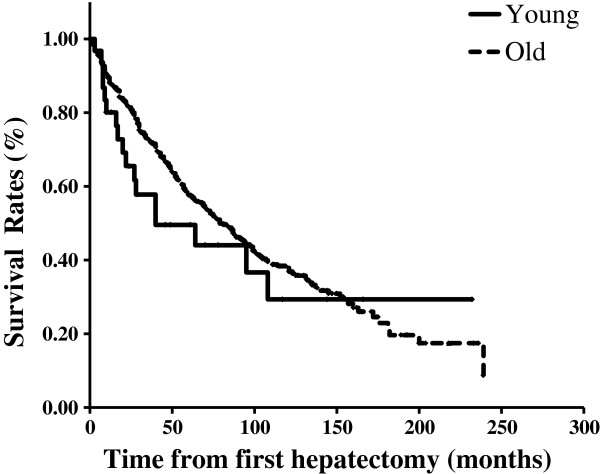
Overall survival curves of the younger and older patient groups after first hepatectomy.

**Figure 2:**
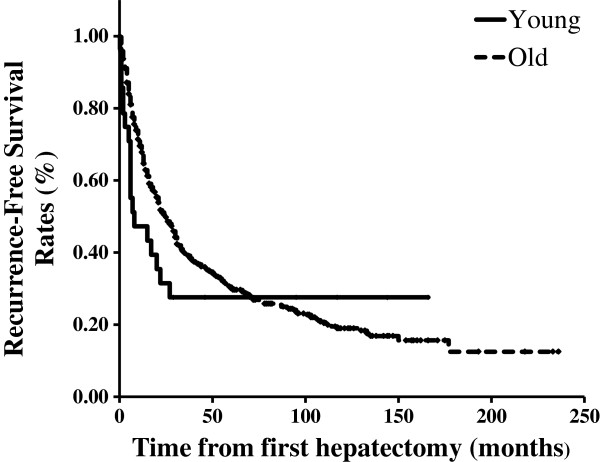
Recurrence-free survival curves of the younger and older patient groups after first hepatectomy.

The five-year OS rate of all 811 patients was 57.1%. The five-year OS rate and median survival time (MST) of the younger group were 49.6% and 40 months, respectively, whereas those of the older group were 57.7% and 79 months, respectively (Figure 1). The median RFS time of all 811 patients was 23 months, while that of the younger patients was 6 months, and that of the older patients was 25 months (Figure 2). Neither OS nor RFS were significantly different between the younger and older groups, although recurrence tended to occur earlier in the younger patients.

### Factors related to long-term survival and disease-free survival after primary hepatectomy in the younger patient group

Table 2 shows those factors that were found by univariate analysis to influence OS and RFS in the younger group. The univariate analysis revealed that OS was significantly related to being HCV-Ab-positive, having a serum albumin level of <4.0 g/l and a maximum tumor size of ≥5 cm, the presence of tumor thrombus in the second and first branches and trunk or opposite side branch of the portal vein (vp2, 3, 4), microscopic intrahepatic metastasis, and histological liver cirrhosis of non-cancerous liver.

**Table 2:** Univariate analyses of prognostic factors of survival and recurrence in the younger group

	**Survival**	**Recurrence**
	***P***	***P***
Epidemiology		
Sex: Male	*NS*	*NS*
HBs-Ag positive	*NS*	*NS*
HCV-Ab positive	0.0172	*NS*
Biochemical Factors		
Albumin <4.0 g/l	0.0088	*NS*
Total bilirubin ≥0.8 mg/dl	*NS*	*Ns*
ICGR15 ≥15	*NS*	*NS*
AFP ≥200 ng/ml	*NS*	*NS*
Tumor Factors		
Number of tumors: multiple	*NS*	0.0199
Maximum size of tumor: ≥5 cm	0.0034	0.0006
Macroscopic classification: except for simple nodular type	*NS*	*NS*
Distant metastasis positive	*NS*	*-*
Surgical Factors		
Non-anatomical resection	*NS*	*NS*
Histological Factors		
Differentiation: poor	*NS*	0.0395
vp2, 3, 4	0.0108	0.0020
im	0.0058	0.0053
cirrhosis	0.0446	*NS*

AFP, alpha-fetoprotein; HBs-Ag, HBs-antigen; HCV-Ab, HCV-antibody; ICGR15, indocyanin green retention rate at 15 minutes; im, microscopic intrahepatic metastasis; NS, non-significant; vp2, tumor thrombus in the second branches of the portal vein; vp3, tumor thrombus in the first branch of the portal vein; vp4, tumor thrombus extension to the trunk or the opposite side branch of the portal vein.

Univariate analysis showed that RFS was significantly related to multiple tumors, maximum tumor size of ≥5 cm, poor differentiation, the presence of tumor thrombus above vp2 and microscopic intrahepatic metastasis. Multivariate analysis showed HCV-Ab-positive status and serum albumin levels of <4.0 g/l to be independent predictive factors for OS, and multiple tumors and vp2, 3, 4 were independent predictive factors for RFS in the younger group of patients (Tables 3 and 4).

**Table 3:** Multivariate analyses of prognostic factors of survival in the younger group

**Risk factor**	***P*-value**	**Hazard ratio**	**95% CI**
HCV-Ab positive	0.0196	59.816	1.927 to 1856.714
Albumin <4.0 g/l	0.0296	6.665	1.207 to 36.813
Maximum size of tumor: ≥5 cm	*NS*	0.381	0.025 to 5.697
vp2, 3, 4	*NS*	2.313	0.420 to 12.738
im	*NS*	14.563	0.951 to 222.939
cirrhosis	*NS*	1.037	0.149 to 7.200

CI, confidence interval; HCV-Ab, HCV-antibody, im, microscopic intrahepatic metastasis; NS, non-signficant; vp2, tumor thrombus in the second branches of the portal vein; vp3, tumor rhombus in the first branch of the portal vein; vp4, tumor thrombus extension to the trunk or the opposite side branch of the portal vein.

**Table 4:** Multivariate analyses of prognostic factors of recurrence in the younger group

**Risk factor**	***P*-value**	**Hazard ratio**	**95% CI**
Number of tumor: multiple	0.0415	51.312	1.163 to 2264.565
Maximum size of tumor: ≥5 cm	*NS*	3.210	0.353 to 29.152
Differentiation: poor	*NS*	2.796	0.450 to 17.043
vp2, 3, 4	0.0253	13.517	1.380 to 132.442
im	*NS*	0.137	0.005 to 3.541

CI, confidence interval; im, microscopic intrahepatic metastasis; NS, non-significant; vp2, tumor thrombus in the second branches of the portal vein; vp3, tumor thrombus in the first branch of the portal vein; vp4, tumor thrombus extension to the trunk or the opposite side branch of the portal vein.

## Discussion

In this study, the younger patients with HCC who underwent hepatectomy were more likely than the older patients to be HBV-positive, to have large tumors with portal invasion and to have high AFP, although they also retained better liver function than the older patients. Despite the significant difference in tumor progression, neither OS nor RFS were significantly different between the two groups, although recurrence tended to occur earlier in the younger patients. Multivariate analysis showed HCV-Ab-positive status and serum albumin levels of <4.0 g/l to be independent predictive factors for OS, and multiple tumors and vp2, 3, 4 were independent predictive factors for RFS in the younger patients. Therefore, young patients with hepatocellular carcinoma should be aggressively treated with hepatectomy due to their good pre-surgical liver function.

In the younger group of patients, HCV-Ab-positive status and low serum albumin levels were the liver-function-related factors that were found to be significantly unfavorable in terms of OS, while multiple tumors and vp2, 3, 4 were the tumor-related factors that were significantly unfavorable in terms of RFS; moreover, these findings were obtained by both univariate and multivariate analyses. Although most of the younger patients had advanced tumors, no differences were found between the younger and older patients in terms of OS. These results indicate that aggressive and curative liver resection should be performed for young patients with HCC, because most young patients retain good pre-surgical liver function.

The definition of who should be classified as a “young patient” with HCC remains controversial. In the literature, the definition of a young patient with HCC has tended to be a patient aged 40 years or younger [4,8,10-12,14]. Cases of HCC in such patients are comparatively rare, for example, HCC occurs in only 0.6 to 2.7% of this age group in Japanese reports [12-14]. In other countries, the reported rates of HCC in this age range are as follows: 8.6% (40 years and younger) in Singapore [11], 10.9% (under 40 years) in Taiwan [8] and 6.5% (40 years and younger) in Hong Kong [4]. Thus most of the existing reports have been from Asia, and they show a difference in frequency among regions. There appear to be many young patients in Asia with HCC who are HBV-positive; HBV is an underlying disease of HCC in young patients, and many carriers live in Asia [17].

Many young patients with HCC have HBs-Ag, that is, up to 71.4 to 100% [3-5,7-11,14]. Meanwhile, cases of HCV-Ab-positivity plus HCC among younger patients are reported at rates of 0 to 10% [4,5,7-10,12,14], which is much lower than the range for older patients. Rates of Child-Pugh A are 69.1 to 92.3% among younger patients [4-6,8-12], which is higher than the range in older patients. It has been reported that histological hepatitis or cirrhosis of non-cancerous liver is significantly less common in younger hepatectomy patients than in older hepatectomy patients among cases with HCC [3,4,12]. Though HCC is generally found by medical examination or follow-up of liver function, in most young patients, HCC is found by symptoms such as pain and/or palpation of an abdominal mass [11,14,18,19]. Accordingly, members of the younger patient group in this study had larger tumors than the older patient group.

This study revealed that the rate of cases related to HBV was 93.5%, and the rate of HBs-Ag-positive cases was 87.0%. The MST of the younger group was 40 months, and the five-year OS rate was 49.6%. These results did not differ significantly from the previously reported MST and five-year OS rates of 27.8 to 52.5 months and 30.5 to 54.8%, respectively, among cases of liver resection for HCC across all ages [20,21]. Therefore, it appears likely that aggressive and curative liver resection contributes to prolonged prognosis.

In regard to tumor factors, several studies have reported that more young than old patients have high AFP levels, that is, the rates of cases in which AFP is equal to or exceeds a value of 400 ng/ml range from 52.6 to 82.0% [3,7,9-11,14], and rates for an AFP of ≥10,000 ng/ml range from 31.6 to 60.0% [3,10,11,14]. In addition, younger patients tend to have larger tumors than older patients, with the maximum diameter of tumors being 6.9 to 12.7 cm in younger patients [3,4,7,10,12,14]. Cases showing portal invasion count for 45.0 to 100% [10-12,14] of younger HCC patients. In the present study, the younger patient group had higher AFP levels and larger tumors, was more likely to have portal invasion and showed better liver function than the older group, as has been reported elsewhere [3,7,10-12,14]. It has also been reported that cases with high AFP levels have a poor prognosis due to a correlation between tumor size and AFP [22].

As regards prognostic factors, Chen *et al.* reported that hepatectomy was a significant favorable prognostic factor among HCC patients aged 40 years and younger [8]. As regards other prognostic factors, AFP [8,11], portal invasion [8,11] and reserved liver function [8,11,12] have been reported, although these remain controversial. In this study, prognostic factors related to OS were HCV-Ab-positive status and low serum albumin levels, and prognostic factors related to RFS were the number of tumors and vp2, 3, 4. It has been suggested that liver function preservation primarily influences survival, and tumor factors influence recurrence. Furthermore, while the time to recurrence in the younger patients was shorter than that in the older patients, the RFS of the younger group tended to overtake that of the older group in the long term. The recurrence rate was 71%, and the site of recurrence was almost always the liver. This rate was comparable to those of other reports, which ranged from 60.2 to 78.2% across all ages [20]. The results to date suggest that aggressive treatments, including re-hepatectomy for recurrence, contribute to an improvement in the long-term prognosis.

Moreover, in order to improve prognosis, we should take care to perform aggressive resections, and should also make note of cases with a background of potentially liver-affecting hepatitis B. Chuma *et al.* reported that the quantity of HBV-DNA and non-treatment for HBV were risk factors for a recurrence of HCC [23]. Li *et al.* reported that one-year and two-year RFS rates were 23.3% vs. 8.3%, and 2.3% vs. 0%, respectively, in a treatment group receiving lamivudine for HCC due to concurrent hepatitis B vs. a control group [24]. Therefore, viral treatments in combination with cancer treatments, including resection, are important to consider.

There have been few reports on liver transplantation for young patients with HCC. The reason for this lack of information is likely to be that younger patients have relatively larger tumors and, therefore, they tend to have tumors exceeding the Milan criteria. Ismail *et al.* reported that the outcomes of liver transplantation were better than those of liver resection among patients with HCC who were aged 2 to 27 years, namely, the OS rates were 72% vs. 40%, and the RFS rates were 91% vs. 30% [25]. It was also reported that primary liver transplantation for children with HCC without extrahepatic lesions has a good outcome, even if the tumors exceed the Milan criteria [26]. An accumulation of future cases is expected.

As noted above, many young HCC patients present with advanced tumors and unfavorable prognostic factors. In a study on 16 patients who received liver transplantation for HCC and who had low differentiation and vascular invasion beyond the Milan criteria, Saab *et al.* reported that those receiving sorafenib (n = 8) had one-year OS rates and RFS rates of 87.5% and 85.7%, versus 62.5% and 57.1% for the control group (n = 8) [27]. It is expected that supportive treatment with molecular target medicine after liver resection or transplantation could contribute to a prolonged prognosis.

## Conclusions

In our younger patients with HCC, survival appeared to be mainly affected by liver function while recurrence was mainly affected by tumor factors. Young patients with HCC should be offered aggressive hepatectomy due to their relatively preserved liver function.

## Abbreviations

AFP: Alpha-fetoprotein; CT: Computed tomography; HBV: Hepatitis B virus; HBs-Ag: HBs-antigen; HCC: Hepatocellular carcinoma; HCV: Hepatitis C virus; HCV-Ab: Hepatitis C virus-antibody; ICGR15: Indocyanin green retention test at 15 minutes; MRI: Magnetic resonance imaging; MST: Median survival time; OS: Overall survival; PIVKA-II: Protein induced by vitamin K absence-II; RFS: Recurrence-free survival; US: Ultrasound sonography; vp2: Tumor thrombus in the second branches of the portal vein; vp3: Tumor thrombus in the first branch of the portal vein; vp4: Tumor thrombus extension to the trunk or the opposite side branch of the portal vein.

## Competing interests

All of the authors declare that they have no competing interests.

## Authors’ contributions

SS carried out the analysis of data and wrote the manuscript. TK and AT gave comments and revised the manuscript. HY, KW, YT, TK and HK made the database of patients. All authors read and approved the final manuscript.
